# Cervical articular process joint versus ventral cervical stabilization in horses: A biomechanical study

**DOI:** 10.1111/vsu.70166

**Published:** 2026-10-05

**Authors:** Machiel P. Ysebaert, Ryan Foslien, Donovan M. David, Andres F. Bonilla, Ben C. Gadomski, Jeremiah T. Easley, Brad B. Nelson

**Affiliations:** ^1^ Colorado State University, College of Veterinary Medicine and Biomedical Sciences Fort Collins Colorado USA; ^2^ Orthopedic Bioengineering Research Laboratory Colorado State University Fort Collins Colorado USA

## Abstract

**Objective:**

To determine the biomechanical properties and modes of failure of a single level equine cervical spine immobilized with unilateral or bilateral articular process joint (APJ) stabilization, ventral cervical stabilization (VCS) and non‐stabilized controls.

**Study design:**

Ex vivo experimental study.

**Sample population:**

A total of 24 equine cadaver cervical spines.

**Methods:**

Cervical spinal units (C4–C5) were randomly assigned to four groups (*n* = 6/group): unilateral APJ stabilization, bilateral APJ stabilization, VCS using pedicle screws and rod constructs, or non‐stabilized. Non‐destructive testing assessed kinematic range of motion (ROM), neutral zone, and stiffness in flexion‐extension, axial rotation, and lateral bending. Specimens underwent four‐point bending to failure (0.1 mm/s). Radiographs and visual inspection characterized failure mode. Groups were compared using one‐way ANOVA or Kruskal‐Wallis tests. Significance was *p* < .05.

**Results:**

Non‐destructive testing showed unilateral and bilateral APJ constructs were stiffer in flexion‐extension, axial rotation and lateral bending than non‐stabilized (*p* < .05). In destructive testing, bilateral APJ stabilization was 2.5× stiffer than VCS, with similar failure moments. Construct stiffness was higher with bilateral APJ stabilization (27.89 ± 5.11 Nm/°) versus non‐stabilized (6.92 ± 3.90 Nm/°, *p* = .0037), unilateral APJ (14.06 ± 2.94 Nm/°, *p* < .0001) and VCS (11.28 ± 3.01 Nm/°, *p* < .0001) groups. Failure typically occurred as cranial articular process fracture in both bilateral and unilateral APJ constructs.

**Conclusion:**

Bilateral APJ stabilization demonstrated greater stiffness than VCS with comparable failure moments.

**Clinical significance:**

Bilateral APJ stabilization, with its higher stiffness than VCS, may be an alternative strategy to achieve spinal fusion in horses while avoiding a ventral surgical approach. Future investigations and development of this technique in vivo are warranted before implementation in clinical cases.

## 1 INTRODUCTION

Cervical vertebral compressive myelopathy (CVCM) is the principal cause of equine non‐infectious spinal ataxia in horses with clinical signs ranging from subtle performance decline to severe neurologic deficits and recumbency.^1^, ^2^, ^3^ Ventral cervical stabilization (VCS) has been the current standard of care to accomplish intervertebral fusion for over 45 years. Additionally, there is increasing evidence that VCS is a viable treatment for horses with other neck disorders (i.e., dynamic spinal nerve impingement) that result in neck pain, stiffness, and radiculopathy that can manifest as dangerous behavior.^4^ Current surgical implants used to achieve VCS include kerf cut cylinders (KCC), locking compression or three‐dimensional (3D) printed plates (with or without spacers), and pedicle screws and rods (PSR).^5^, ^6^, ^7^, ^8^ Despite advancements in implants, all require a ventral surgical approach and deep dissection to access the cervical vertebrae.^9^ The ventral approach risks damage to critical neurovascular structures, the trachea and esophagus. Alternative approaches to stabilize the cervical spine beyond the conventional ventral approach are indicated.

In human spinal surgery, similar complications observed in horses have driven investigation to alternative strategies to accomplish vertebral stabilization including articular process joint (APJ) stabilization.^10^, ^11^ There are three articulations between adjoining vertebra: ventrally through the intervertebral disc and dorsally by two APJ connections. APJ stabilization (referred to as the translaminar facet technique in human surgery) provides biomechanically equivalent resistance as the PSR construct,^12^ and translaminar facet stabilization showed improved patient pain scores.^13^ Biomechanical characterization of APJ stabilization, however, has been scarcely published in small animals,^14^, ^15^, ^16^ ^,^ and is under preliminary investigation in horses.^17^ Translating these methods to horses with cervical dysfunction is compelling. However, equine vertebral anatomy has notable differences from dogs and humans. Determining kinematic motion and stability after APJ stabilization and as it compares to conventional VCS is prudent prior to its investigation in horses with clinical disease.

The objective of this study was to investigate biomechanical features of APJ stabilization at a single cervical vertebral segment in the horse, specifically as it compares to VCS and non‐stabilized controls. We hypothesized that unilateral and bilateral APJ stabilization would have increased stiffness compared to non‐stabilized controls, and that bilateral cervical APJ stabilization would not have different stiffness compared to VCS.

## 2 MATERIALS AND METHODS

### 2.1 Study design and specimen preparation

Equine cervical vertebral columns were obtained postmortem from horses presenting to the Johnson Family Equine Hospital at Colorado State University that were euthanized for reasons unrelated to this study. Clients provided written consent for their horses to be used in research. A total of 25 cervical spines were collected within 24 h and stored at −20°C. One spine was used for a preliminary study, while 24 spines were used for the primary study. Cervical spines were disarticulated at the C3–C4 and C5–C6 levels: isolating C4–C5 for testing. The C4–5 level was chosen due to its standard use in biomechanical stabilization studies thereby enabling qualitative comparison to prior studies.^18^, ^19^, ^20^ The external musculature was removed (including the fibrous capsules of the APJs). The APJ cartilage and intervertebral disc remained intact. After soft tissue removal, the cervical spinal units were placed in sealed plastic bags and stored at −20°C until the time of implant placement and biomechanical testing. The specimens were thawed at room temperature for 12 h. Four freeze–thaw cycles from collection to completion of biomechanical testing occurred and specimens were not subjected to >20 h at room temperature.

### 2.2 Surgical implantation

All surgical procedures were completed by the same surgical resident (MPY) under the direct supervision of a board‐certified equine surgeon (BBN). Each cervical spinal unit was randomly assigned after thawing to one of four groups (6/group): (1) Unilateral APJ stabilization, (2) bilateral APJ stabilization, (3) VCS using PSR, or (4) non‐stabilized. Radiographs were performed pre‐ and post‐implantation of each specimen to verify no pre‐existing disease or iatrogenic damage after implant placement. Radiographic projections included dorso‐ventral, lateral, left 45° ventro‐lateral right dorso‐lateral oblique and left 45° dorso‐lateral right ventro‐lateral oblique views (Figure 1).

**FIGURE 1:**
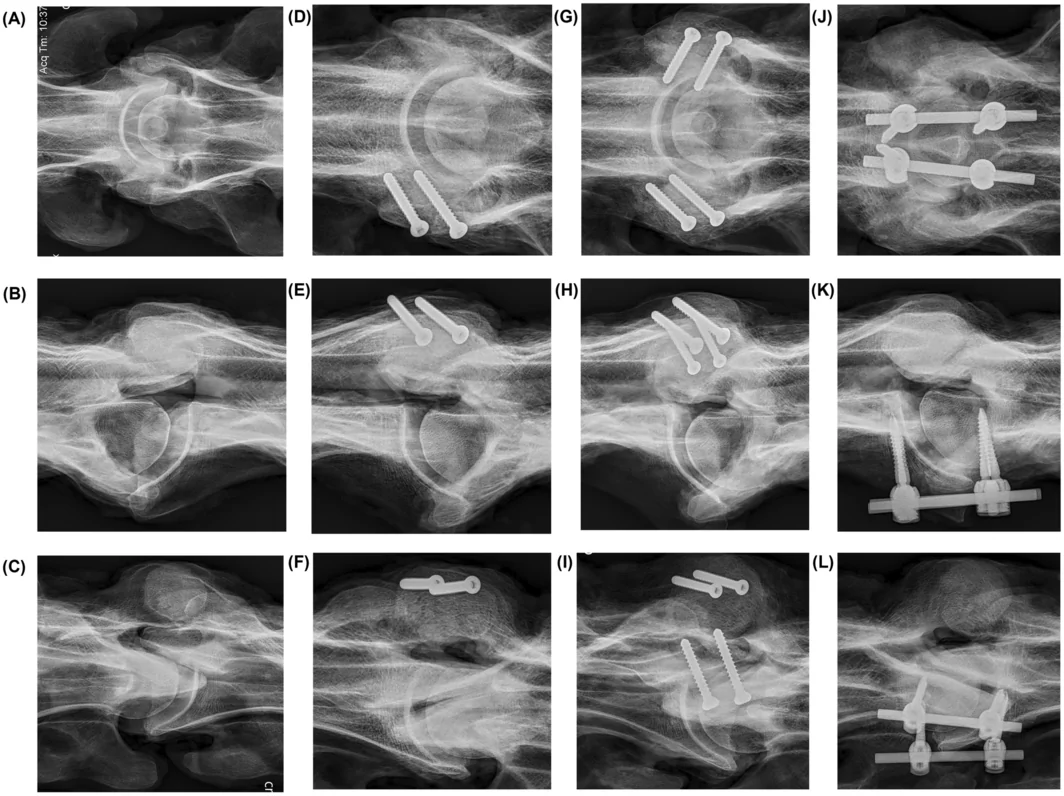
Radiographic images of the four experimental groups. Non‐stabilized group (A–C), unilateral articular process joint (APJ) stabilization group (D–F), bilateral APJ stabilization group (G–I), and ventral cervical stabilization group (J–L). Cranial is to the left and caudal to the right. Rows correspond to radiographic projections: First row, dorsoventral; second row, laterolateral; third row, 45° ventrolateral‐right dorsolateral oblique.

### 2.3 Preliminary study

Prior to testing experimental groups, a trial experiment was performed to optimize the biomechanical testing protocols and to corroborate the screw size used for unilateral and bilateral APJ stabilization. Two cervical spinal units (C2–C3 and C4–C5) from the same horse were isolated. The C2–3 segment was stabilized with four 5.5 × 38 mm cortex screws (2 screws/APJ), and the C4–C5 segment with four 4.5 × 38 mm cortex screws placed using lag technique perpendicularly across the APJs in 45° ventro‐abaxial‐caudal to dorso‐axial‐cranial direction. Once all four screws were placed, they were tightened using a 4 Nm torque limiting device (Synthes, Centennial, Colorado). Non‐destructive and destructive testing were performed to assess biomechanical features and failure modes between screw sizes.

### 2.4 Primary study

#### 2.4.1 Unilateral APJ stabilization

Our rationale for including unilateral APJ stabilization was primarily investigatory. We sought to determine whether unilateral stabilization could provide sufficient stability while likely reducing anesthesia and procedure time of bilateral stabilization. For unilateral APJ stabilization, two 5.5 mm cortex screws were placed using lag technique across one APJ; the contralateral APJ was non‐stabilized. The side was randomly assigned by a coin toss, using a balanced design. The screw size was selected based on the results of the preliminary study. Two 5.5 mm glide holes were made 10 mm abaxial to the most axial aspect of the C5 cranial articular process and spaced at 1/3 of its total width (measured by ruler: 10–15 mm). The drilling trajectory was approximately 45° ventro‐abaxial‐caudal to dorso‐axial‐cranial direction to traverse the APJ surface perpendicularly. The process was repeated for the second glide hole. A 5.5 mm centering sleeve was placed into the first glide hole, and a 4.0 mm thread hole was drilled across the caudal C4 articular process. Routine countersinking (using 3 progressive quarter turns), depth gauging and tapping was performed prior to 5.5 mm cortex screw placement. The first screw was placed but not fully tightened to prevent uneven compression across the APJ. Then, the second thread hole was drilled, followed by countersinking, depth gauge measurement, tapping and the second screw placed. A bone reduction forceps was not used as it did not reliably provide even compression across the APJ. After both screws were placed, they were both tightened using a 4 Nm torque limiting device (Synthes, Centennial) (Figure 1). Screw lengths ranged from 34 to 40 mm ensuring that threads engaged the far cortex of the APJ.

#### 2.4.2 Bilateral APJ stabilization

The same screw placement protocol was used for bilateral APJ stabilization as unilateral APJ stabilization, though both APJs were compressed with two 5.5 mm cortex screws on the left side before repeating the process on the right side (Figure 1). After screws were placed across both APJs, all four screws were tightened using a 4 Nm torque limiting device (Synthes, Centennial). In the rare instance, screw heads were to sink into the bone prior to reaching 4 Nm, further tightening was stopped. Screw lengths also ranged from 34 to 40 mm.

#### 2.4.3 Ventral cervical stabilization (VCS) with pedicle screws and rods (PSR)

For the VCS group, the polyaxial pedicle screws were placed under fluoroscopic guidance to ensure the intervertebral disc was not entered. Two 5.5 mm diameter × 30 mm length polyaxial titanium self‐tapping pedicle screws (ArteMedics) with dual core screw bodies were placed in the vertebral bodies caudally in C4, and two were placed cranially in C5. A 3.2 mm drill bit was used to create unicortical holes into the vertebral body. Then, the self‐tapping polyaxial pedicle screws were advanced into the pilot hole until the swiveling head of the screw reached the cortical surface but was still able to move multi‐directionally. After all four screws were placed, two 5.5 mm diameter titanium rods (80 mm length; ArteMedics) were placed into the pedicle screw heads, spanning the disc on either side of midline. Set screws were tightened, locking the rods and screw trajectories in place using a custom torque limiter (ArteMedics). Final radiographic images were taken to ensure proper pedicle screw placement (Figure 1).

#### 2.4.4 Non‐stabilized control

For the control group, the synovial capsule was bilaterally removed and no implants were placed.

### 2.5 Non‐destructive testing

Non‐destructive biomechanical kinematic testing was performed to determine range of motion (ROM), neutral zone and stiffness at C4–C5 of each specimen. Methods were replicated from comparable ex vivo studies testing VCS stabilization methods.^18^, ^21^ Each spinal unit was fixed in hard cast resin (SmoothCast 320, Smooth‐On, Macungie, Pennsylvania) on cranial C4 and caudal C5. Specimens were kept hydrated with 0.9% saline to prevent desiccation during testing. A custom‐built testing system applied 6.0 Newton meters (Nm) of force in three principal directions (flexion–extension, lateral bending, and axial rotation) using a 6 degrees of freedom load transducer (MC3‐6‐1K, Advanced Mechanical Technology, Inc., Waterton, Massachusetts) to measure applied moments. A four‐camera stereophotogrammetry system (Motion Analysis Corporation, Santa Rosa, California) tracked optical marker triads anchored to each spinal unit to measure relative spinal motion in three‐dimensions, and the related Euler angles for the relative motion were calculated. Five cycles of non‐destructive loading in flexion‐extension, lateral bending, and torsion provided preconditioning; The fifth data cycle collected from each testing direction was used for reported outcomes. Parameters were measured and calculated for each loading mode. The parameters of interest were ROM (°), elastic and neutral zone stiffness (Nm/°), and neutral zone (°). ROM was calculated as the absolute difference between the maximum and minimum angles measured during the last loading cycle. Elastic zone stiffness was calculated as the inverse of the slope of the moment‐ROM curve over the elastic region. Neutral zone was calculated as the magnitude of motion (in °) in the region of the relatively low stiffness with limits defined by maxima and minima of the second derivative of the ROM curve.^22^, ^23^ Neutral zone stiffness was calculated as the inverse of the slope of the moment‐ROM curve over the neutral zone region.^24^

### 2.6 Destructive testing

All specimens were examined after non‐destructive testing to ensure constructs were undamaged prior to destructive testing. The spinal units were then loaded until failure in flexion (MTS 370 Landmark, MTS Systems Corporation, Eden Prairie, Minneapolis, Minnesota). A steel four‐point bending fixture applied a controlled constant displacement at 0.1 mm/s to failure (Figure 2).^18^ Actuator displacements and applied loads were recorded. Digital image analysis was used to measure the change in distance of the contact points, and force and distance data were used to calculate the peak moment at the point of failure. Stiffness was calculated as the peak moment divided by the change in angle during the test. Radiographs and visual inspection were performed after destructive testing to characterize the mode of failure.

**FIGURE 2:**
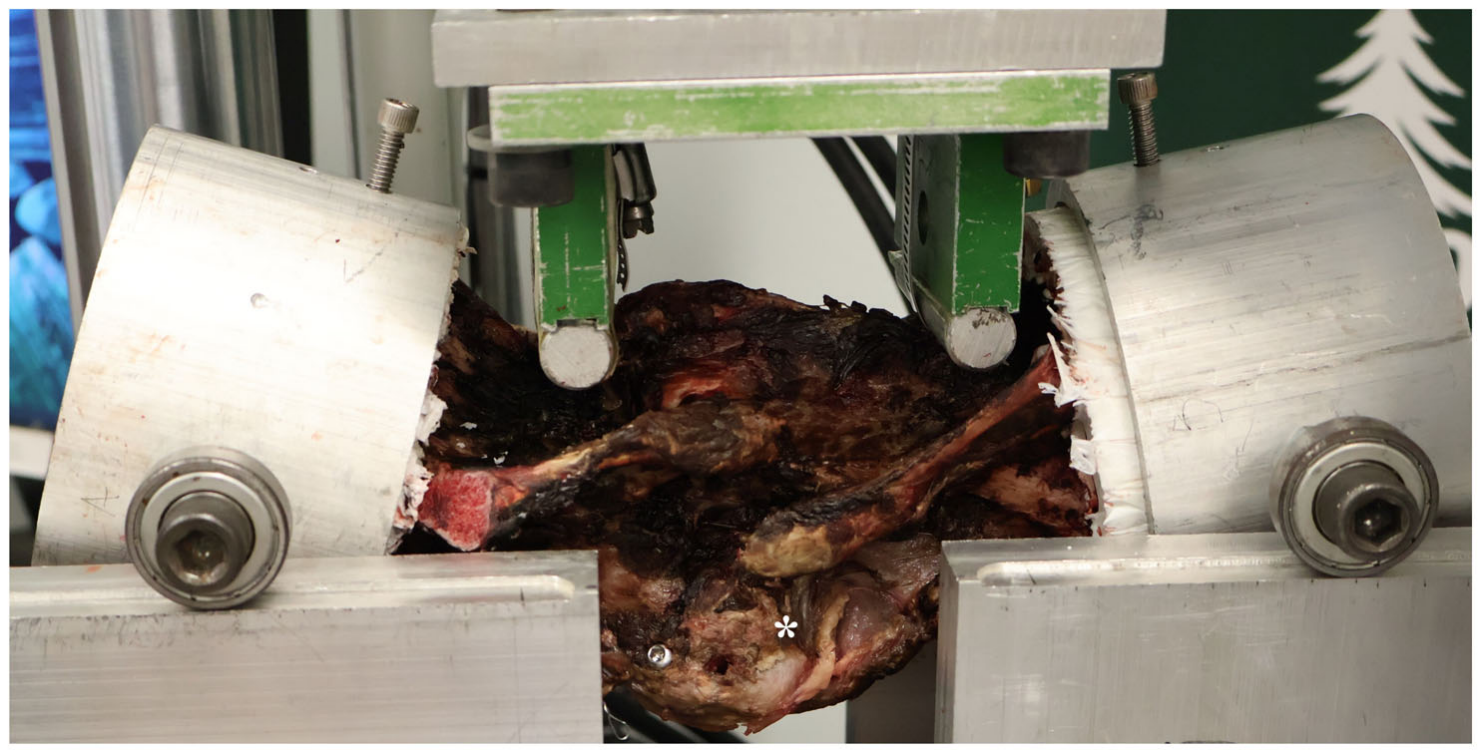
Steel four‐point bending. Cervical spinal unit (C4–5) in flexion: Post‐destructive testing. Asterisk, left articular process joint; cranial is to the right.

### 2.7 Data and statistical analysis

All quantitative data are reported as mean ± SD. Shapiro–wilk testing and visual assessment of quantile‐quantile plots were used to assess data normality. A one‐way ANOVA was used to determine differences in measurement outcomes between the four groups for both non‐destructive and destructive testing outcome measures. Data that was not normally distributed, underwent non‐parametric testing using a Kruskal‐Wallis one‐way ANOVA. Model assumptions were verified. Unequal variances were accounted for using Welch's test. Multiple testing corrections were performed using Tukey's (ANOVA) or Dunn's (Kruskal Wallis ANOVA) adjustment. Statistical analyses were performed using GraphPad Prism (version 10.4.1., Boston, Massachusetts). Significance was defined at *p* < .05.

### 2.8 Sample size and power calculation

The sample size calculations were generated from published papers and our unpublished biomechanical data using G*Power (version 3.1) using our primary outcome of interest (stiffness).^6^, ^18^ Stiffness of the VCS explanted cervical spine using PSR is 29.5 ± 7.6 Nm/° and a non‐stabilized control is 17.4 ± 4.2 Nm/°. With an effect size of 2, and a type I error rate of 0.5 and 0.8 power, six samples per group were needed to detect a difference between groups.

## 3 RESULTS

### 3.1 Specimen demographics

Breeds of the collected cervical spines for the primary study included Quarter Horse (*n* = 15), Thoroughbred (*n* = 5), Warmblood (*n* = 3), and Arabian (*n* = 1). The cadavers were composed of 13 geldings, nine mares and two stallions aged 15.5 ± 8.3 years old (range: 2–28 years) and weighed 471.5 ± 102.2 kg. Pre‐implantation radiographs and visual inspection confirmed no visible abnormalities to the intervertebral discs, vertebral bodies or APJs.

### 3.2 Preliminary study

Non‐destructive testing showed 1.5x higher stiffness for APJ stabilization using 5.5 mm screws compared with 4.5 mm screws. The 5.5 mm screws demonstrated higher ultimate failure point (i.e. peak moment) than 4.5 mm screws (Table 1).

**TABLE 1:** Preliminary study: Destructive testing: Higher ultimate failure point (peak moment) for bilaterally stabilized APJ with 5.5 mm cortex screws when compared with bilaterally 4.5 mm cortex screw stabilization.

Destructive and non‐destructive testing				
Bilateral APJ stabilization	Levels	Peak moment (Nm)	Stiffness (Nm/°)	Compliance (°/Nm)
4.5 mm cortex screw	C4–C5	384	34	0.0294
5.5 mm cortex screw	C2–C3	423	51	0.0196

*Note*: Non‐destructive testing: A higher stiffness (Nm/°) and lower compliance (°/Nm) for the bilaterally 5.5 mm stabilized APJs when compared to the bilaterally 4.5 mm APJ stabilization.

Abbreviations: APJ, articular process joint; °, degrees; Nm, Newton meters.

The mode of failure when using 4.5 mm screws was a combination of screw bending and pullout (Figure 3). The 5.5 mm screw group failed by fracture of the caudal articular process, and no bending or pullout of the screws was observed (Figure 3).

**FIGURE 3:**
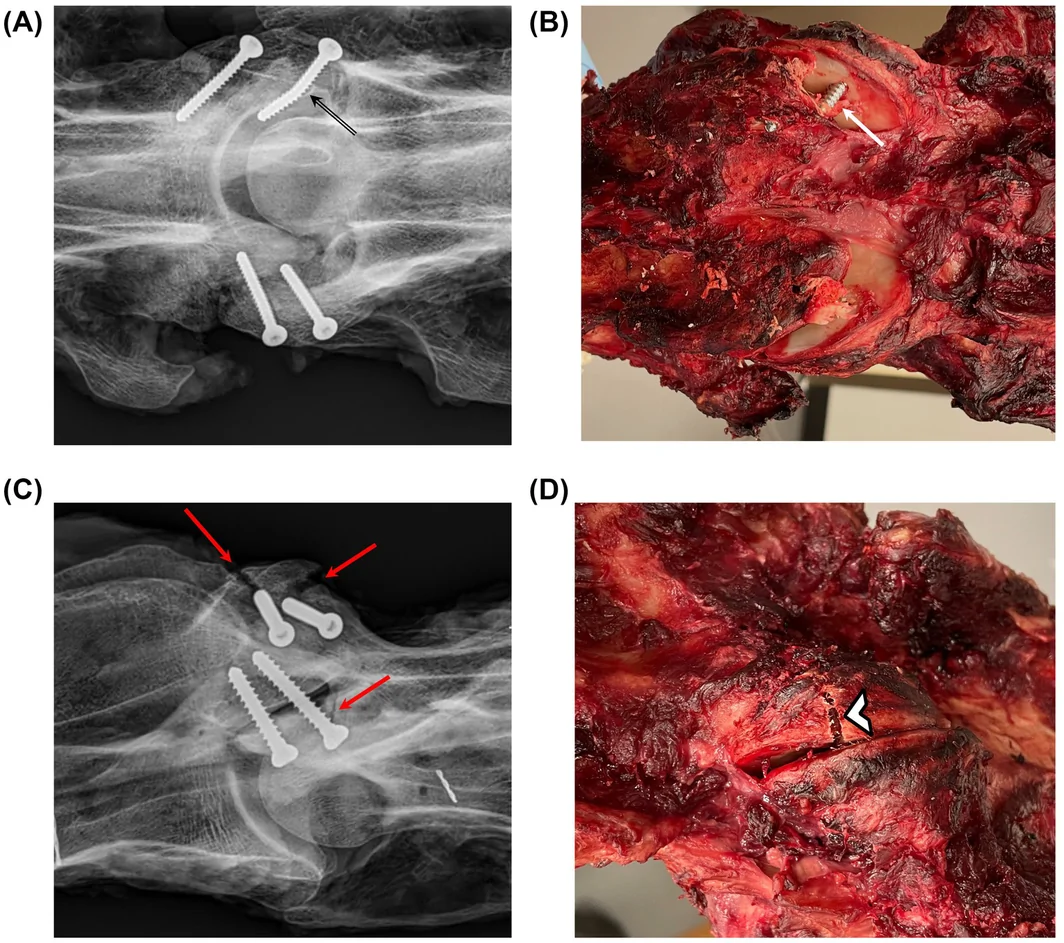
Preliminary study. Radiograph and image of a bilaterally stabilized articular process joint (APJ) cervical unit (C4–5) post‐testing using four 4.5 × 38 mm cortex screws placed in lag (A and B, respectively). Radiograph and image of a bilaterally stabilized APJ cervical unit (C2–3) post‐testing using four 5.5 × 38 mm cortex screws placed in lag (C and D, respectively). Cranial is to the left and caudal to the right. Dorso‐ventral view: Implant failure (black arrow) (A). Dorsal view: 4.5 mm cortex screw pull‐out of the right caudal articular process of C4 (white arrow) (B). Left 45°ventro‐lateral‐right dorso‐lateral oblique view: Mode of failure (red arrows, fracture lines of the APJs) (C). Left APJ of C2–C3: The fracture line in the caudal articular process of C4 (arrowhead) (D).

### 3.3 Flexion‐extension

In flexion‐extension (Figure 4), ROM was lowest in the bilateral APJ stabilization group (0.31 *±* 0.23°). The bilateral APJ group was lower than the non‐stabilized group (20.19 *±* 4.07°, *p* = .0001) and the VCS group (2.54 *±* 1.10°, *p* = .0374). ROM in the unilateral APJ group (0.67 *±* 0.35°) was lower than the non‐stabilized group (*p* = .0132). There was no difference in ROM between bilateral APJ and unilateral APJ stabilization groups (Figure 4B).

**FIGURE 4:**
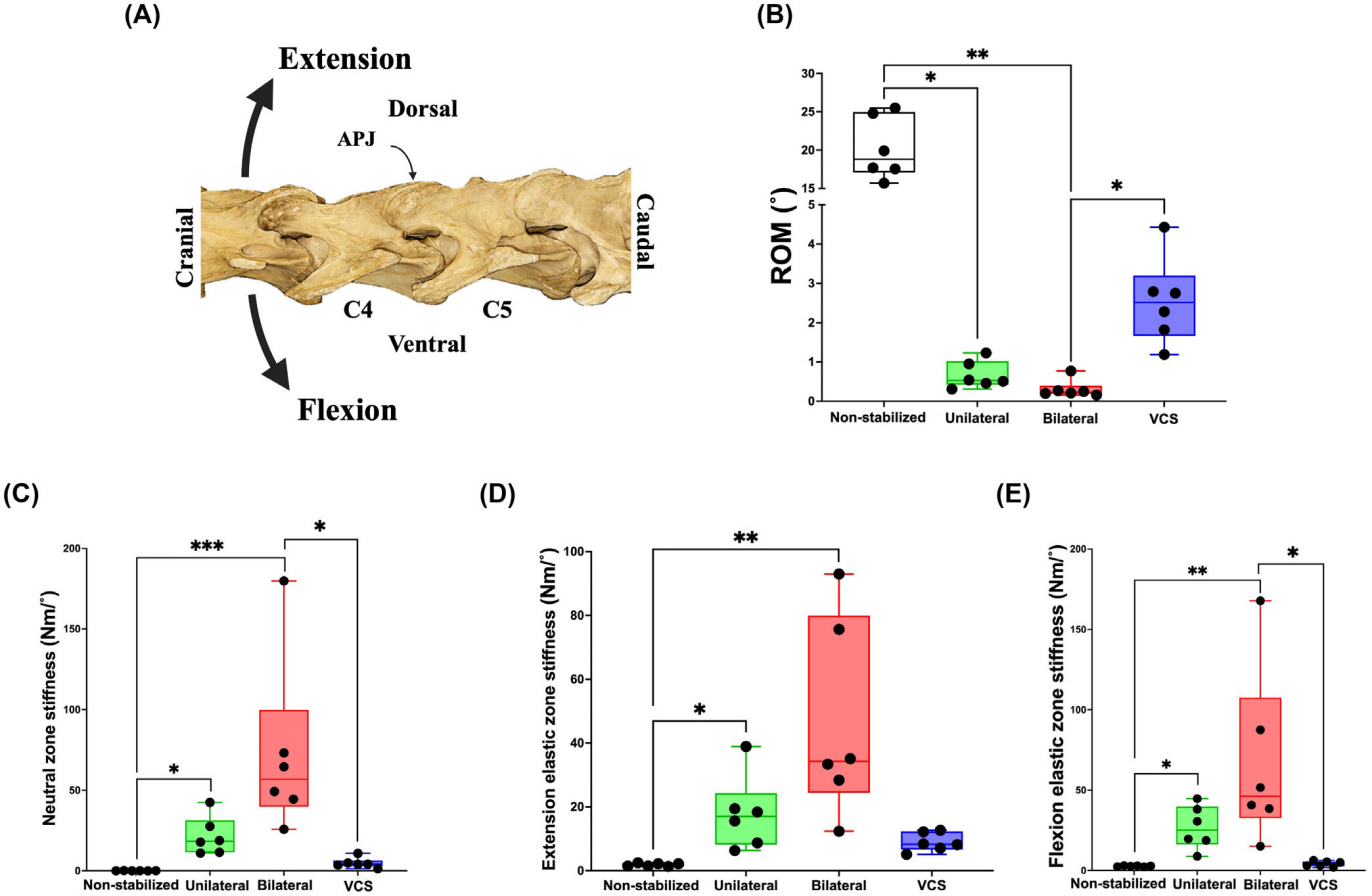
Non‐destructive testing flexion‐extension. Illustration of testing, cervical spine viewed from left lateral (A). Boxplots show mean and SD with minimum/maximum values; circles are individual data points. Range of motion (ROM) (B), Neutral zone stiffness (C), Extension elastic zone stiffness (D), and Flexion elastic zone stiffness (E) results for non‐stabilized control, unilateral articular process joint (APJ) stabilization, bilateral APJ stabilization and ventral cervical stabilization (VCS) groups. *p < .05, **p < .001, ***p < .0001. C4, fourth cervical vertebra; C5, fifth cervical vertebra; Nm, Newton meters.

Neutral zone stiffness was highest in the bilateral APJ stabilization group (72.86 ± 54.94 Nm/°). The bilateral APJ stabilization group and unilateral APJ stabilization group (21.56 *±* 11.88 Nm/°) were higher than the non‐stabilized group (0.10 *±* 0.06 Nm/°) (*p* < .0001 and *p* = .0151, respectively). The bilateral APJ stabilization group was stiffer in neutral zone than the VCS group (4.84 *±* 3.17 Nm/°, *p* = .0256). There was no difference in neutral zone stiffness between the bilateral APJ and unilateral APJ stabilization groups (Figure 4C).

Elastic zone stiffness in extension was highest in the bilateral APJ stabilization group (46.31 *±* 31.00 Nm/°). Elastic zone stiffness in extension of the bilateral APJ stabilization group and unilateral APJ stabilization group (17.90 *±* 11.56 Nm/°) were higher than the non‐stabilized group (1.86 *±* 0.49 Nm/°) (*p* = .0003 and *p* = .0197, respectively). Elastic zone stiffness in extension of the VCS group (8.94 *±* 2.95 Nm/°) was not different than the other groups. There was no difference between bilateral APJ and unilateral APJ stabilization groups (Figure 4D).

Elastic zone stiffness in flexion was highest in the bilateral APJ stabilization group (66.91 *±* 54.80 Nm/°). Elastic zone stiffness in flexion of the bilateral APJ stabilization group and unilateral APJ stabilization group (26.78 *±* 13.46 Nm/°) were higher than the non‐stabilized group (2.72 *±* 0.34 Nm/°) (*p* = .0007 and *p* = .0197, respectively). Elastic zone stiffness in flexion of the bilateral APJ stabilization group was higher than the VCS group (4.17 *±* 1.69 Nm/°, *p* = .0197) (Figure 4E).

### 3.4 Axial rotation

In axial rotation (Figure 5), ROM was lowest in the bilateral APJ stabilization group (0.14 *±* 0.11°). The bilateral APJ group had lower ROM than the non‐stabilized group (1.94 *±* 0.96°, *p* = .0002) (Figure 5B). No difference was observed among the other groups.

**FIGURE 5:**
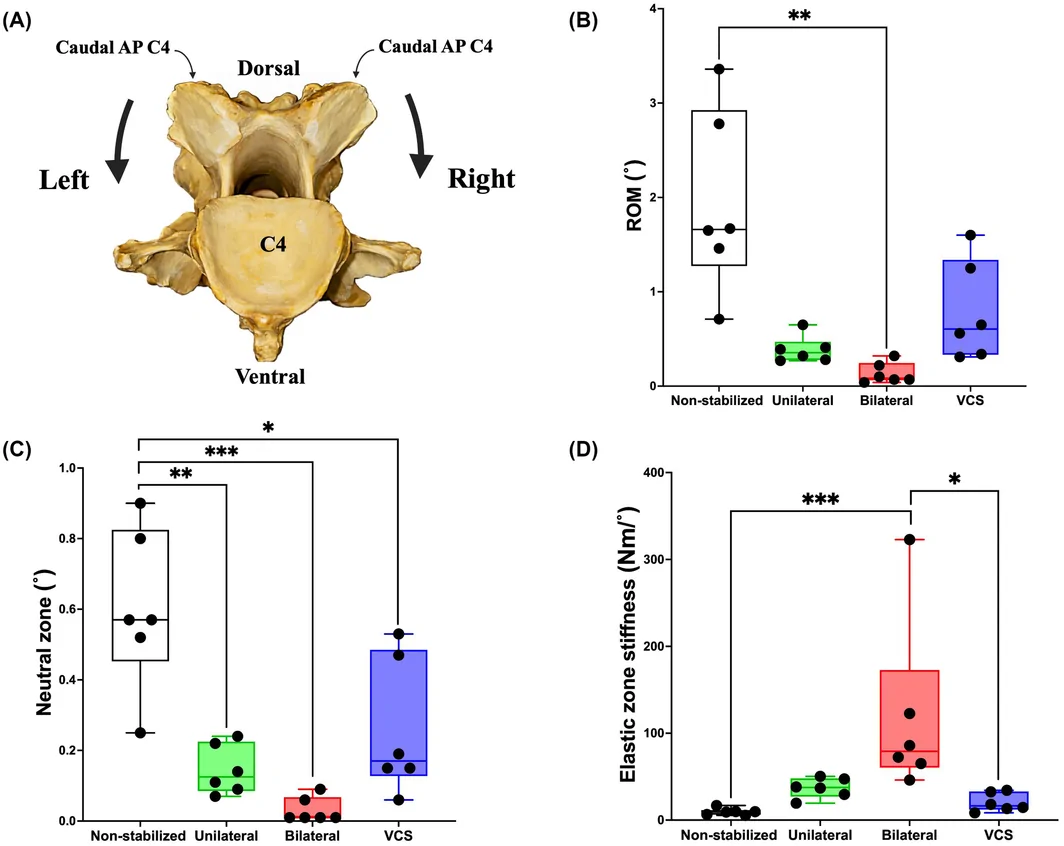
Non‐destructive testing axial rotation. Illustration of testing, cervical spine viewed from caudal (A). Boxplots show mean and SD with minimum/maximum values; circles are individual data points. Range of motion (ROM) (B), Neutral zone stiffness (C), and Extension elastic zone stiffness (D) results for non‐stabilized control, unilateral articular process joint (APJ) stabilization, bilateral APJ stabilization and ventral cervical stabilization (VCS) groups. *p < .05, **p < .001, ***p < .0001. Caudal AP C4, caudal articular process of the fourth cervical vertebra; Nm, Newton meters.

Neutral zone was lowest in the bilateral APJ stabilization group (0.03 ± 0.03°) (Figure 5C). Neutral zone of the non‐stabilized group (0.6 *±* 0.23°) was higher when compared to all groups (bilateral APJ: *p* < .0001; unilateral APJ: 0.14 ± 0.07°, *p* = .0003; VCS: 0.26 ± 0.19°, *p* = .0197). There was no difference in neutral zone among bilateral APJ stabilization, unilateral APJ stabilization and VCS groups.

Elastic zone stiffness was highest in the bilateral APJ stabilization group (119.21 ± 103.01 Nm/°). Elastic zone stiffness of the bilateral APJ stabilization group was higher than the non‐stabilized (9.59 ± 3.95 Nm/°) and the VCS (20.21 ± 10.68 Nm/°) group (*p* = .0003 and *p* = .0197, respectively). There was no difference in elastic zone stiffness between the unilateral (37.00 ± 11.37 Nm/°) and bilateral APJ groups (Figure 5D).

### 3.5 Lateral bending

In lateral bending (Figure 6), ROM was lowest in the bilateral APJ group (0.09 *±* 0.03°). ROM in the bilateral APJ stabilization group was lower than the VCS group (4.64 *±* 2.85°) and non‐stabilized group (21.31 *±* 3.07°) (*p* = .0305 and *p* = .0001, respectively). ROM in the unilateral APJ group (0.66 *±* 0.23°) was lower than the non‐stabilized group (*p* = .0130). There was no difference in ROM between bilateral APJ and unilateral APJ groups (Figure 6B).

**FIGURE 6:**
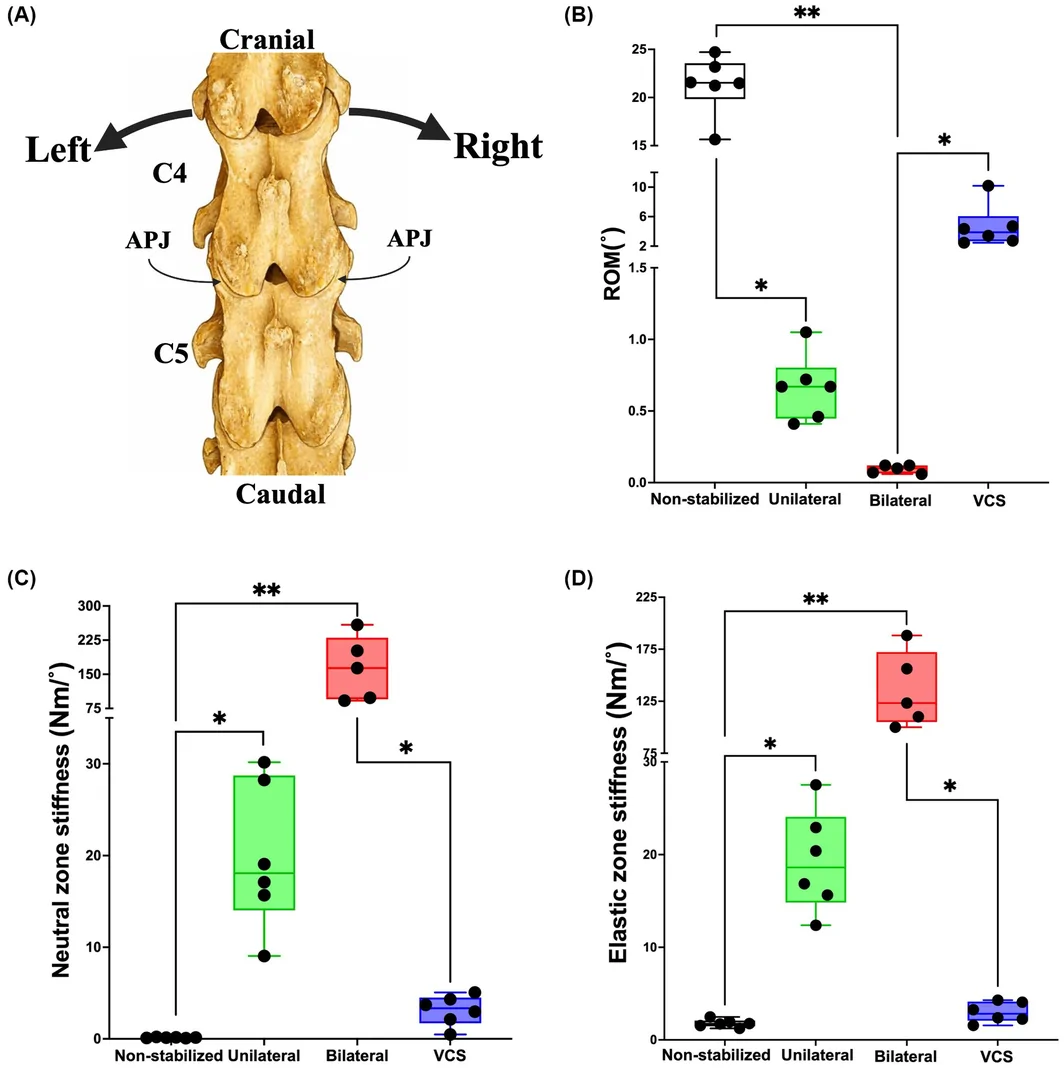
Non‐destructive testing lateral bending. Illustration of testing, cervical spinal unit viewed from dorsal (A). Boxplots show mean and SD with minimum/maximum values; circles are individual data points. Range of motion (ROM) (B), Neutral zone stiffness (C), and Elastic zone stiffness (D) results for non‐stabilized control, unilateral articular process joint (APJ) stabilization, bilateral APJ stabilization and ventral cervical stabilization (VCS) groups. *p < .05, **p < .001. C4, fourth cervical vertebra; C5, fifth cervical vertebra; Nm, Newton meters.

Neutral zone stiffness was highest in the bilateral APJ stabilization group (162.86 ± 70.45 Nm/°). Neutral zone stiffness of the bilateral APJ stabilization group was higher than non‐stabilized (0.14 *±* 0.05 Nm/°, *p* = .0001) and VCS groups (3.11 *±* 1.65 Nm/°, *p* = .0306). The unilateral APJ group (19.88 *±* 7.99 Nm/°, *p* < .05) was stiffer than the non‐stabilized group (Figure 6C). There was no difference between the bilateral and unilateral APJ stabilization groups.

Elastic zone stiffness was highest in the bilateral APJ stabilization group (135.55 ± 36.28 Nm/°). Elastic zone stiffness in the bilateral APJ stabilization group was higher than non‐stabilized (1.78 ± 0.42 Nm/°) and VCS groups (2.97 ± 1.09 Nm/°) (*p* = .0004 and *p* = .014, respectively). The unilateral APJ group (19.28 ± 5.46 Nm/°) was higher than non‐stabilized controls (*p* = .0298). There was no difference between bilateral APJ and unilateral APJ groups (Figure 6D).

### 3.6 Destructive testing

Construct stiffness (Figure 7), was higher in the bilateral APJ stabilization (27.89 ± 5.11 Nm/°) when compared with the non‐stabilized (6.92 ± 3.90 Nm/°, *p* = .0037), unilateral APJ (14.06 ± 2.94 Nm/°, *p* < .0001) and VCS (11.28 ± 3.01 Nm/°, *p* < .0001) groups. The unilateral APJ group was stiffer than the non‐stabilized group (*p* = 0.0205). There was no difference in construct stiffness between the unilateral APJ stabilization and VCS groups (Figure 7B).

**FIGURE 7:**
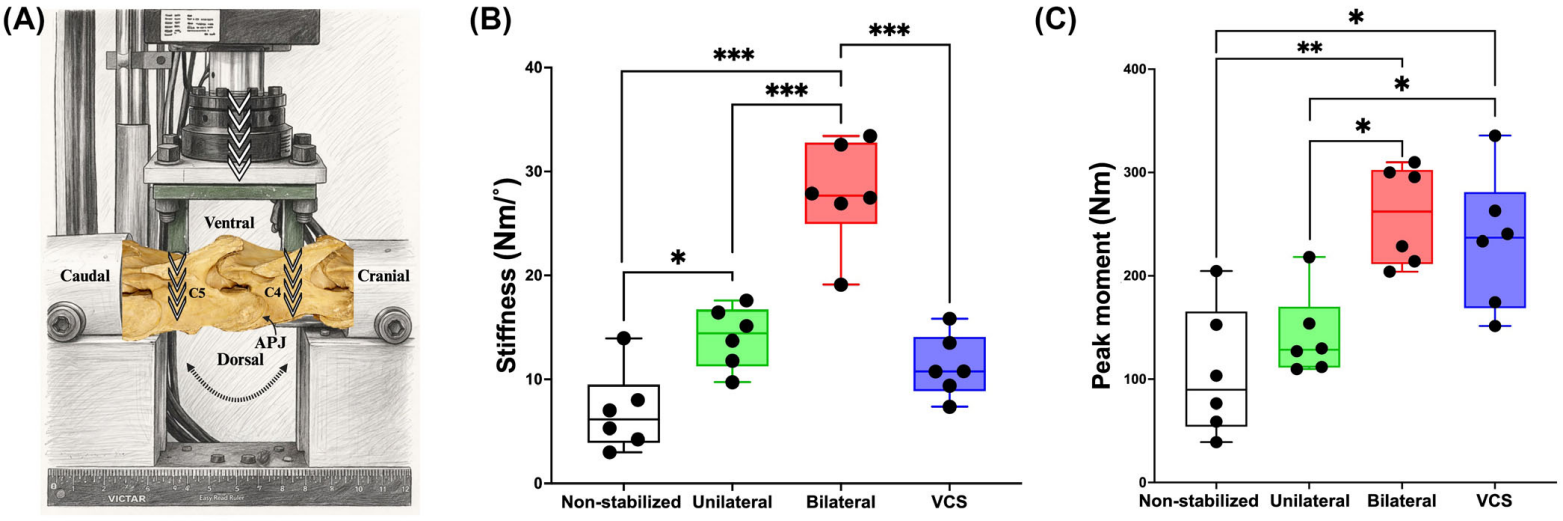
Destructive testing. Illustration of four‐point bending to failure (A). Box plots show mean and SD with minimum/maximum values; circles are individual data points. Stiffness (B) and Peak moment to failure (C) results for non‐stabilized control, unilateral articular process joint (APJ) stabilization, bilateral APJ stabilization and ventral cervical stabilization (VCS) groups. *p < .05, **p < .001, ***p < .0001. C4, fourth cervical vertebra; C5, fifth cervical vertebra. Arrowheads, forces applied; dashed arrow: movement of cervical vertebrae; Nm, Newton meters.

The peak moment at failure of the bilateral APJ stabilization group (258.70 ± 48.04 Nm) was higher than the unilateral APJ stabilization group (141.79 ± 40.59 Nm) and the non‐stabilized group (105.9 ± 62.4 Nm) (*p* = .0006 and *p* = .0076, respectively). The peak moment at failure for the VCS group (233.03 ± 65.63 Nm), however, was not different when compared with the bilateral APJ stabilization group. The unilateral APJ group was lower than the bilateral APJ stabilization (*p* = .0076) and the VCS group (*p* = .0435) (Figure 7C).

### 3.7 Mode of failure

All constructs in unilateral and bilateral APJ stabilization groups failed by bone fracture through the cranial or caudal articular process (AP). Fracture of the cranial AP of C5 was most often observed in the unilateral APJ stabilization group (5/6) and in the bilateral APJ stabilization group (5/6). Cranial AP fractures originated from the caudal glide hole propagating laterally with breakout at the lateral aspect of the AP (Figure 8A,B). Additionally, the fracture line propagated towards the cranial glide hole with breakout laterally to the cranial glide hole creating a lateral osseous wedge fragment (Figure 8C–E). One cervical unit in the unilateral APJ stabilization group failed by a fracture line originating from the cranial glide hole of the cranial AP of C5 and propagating laterally and cranially, creating a wedge fragment at the cranio‐lateral aspect. Additionally, one cervical unit in the bilateral APJ stabilization group fractured from the caudal glide hole of the cranial AP of C5, though propagated caudally, creating a wedge fragment at the caudal aspect. Failure through fracture of the caudal AP of C4 occurred in the unilateral APJ stabilization group (1/6) and bilateral APJ stabilization (1/6) groups.

**FIGURE 8:**
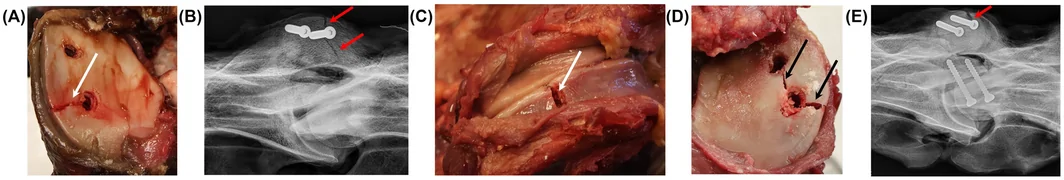
Mode of failure. Unilateral articular process joint (APJ) stabilization (A–B). Dorsal aspect of a cranial articular processes after disarticulation depicting the fracture line (white arrow) (A). Radiographic latero‐lateral view with fracture line (red arrows) (B). Bilateral APJ stabilization (C–E). Lateral view of the right APJ: Fracture line of the cranial articular process (white arrow) (C). Dorsal aspect cranial articular processes after disarticulation depicting the fracture line (black arrows) on the right cranial articular process (D). Radiographic latero‐lateral view with fracture line (red arrows) (E).

The mode of failure for the non‐stabilized group (6/6) and the VCS group (5/6) was most often by dorsal luxation of the APJs. One cervical unit in the VCS group failed by fracturing through the vertebral body of C5. The fracture originated at the caudal screws of the construct; however, no overt fracture line could be observed on radiographic views.

## 4 DISCUSSION

These findings demonstrated that the unilateral and bilateral APJ stabilization groups had increased stiffness compared to non‐stabilized controls and thus supported our first hypothesis. Second, we hypothesized that bilateral APJ stabilization would not have a different stiffness than the VCS group. In non‐destructive testing, the neutral zone stiffness in flexion of the bilateral APJ stabilization group was higher, and the range of motion was lower when compared to the VCS group. Moreover, in destructive testing bilateral APJ stabilization was about 2.5× stiffer than VCS with a comparable but higher moment of failure. Thus, the bilateral APJ stabilization was stiffer than the VCS group, which did not support our second hypothesis.

With the advent of imaging‐guided and computer‐assisted surgery and emerging surgical implants, there may be new opportunities to revisit how cervical stabilization has been conventionally performed in horses.^25^ Application of bone plates on the ventral aspect of cervical vertebrae violates the ideal situation of placing bone plates on the tension side of a bone; however, it is still considered ideal for cervical stabilization due to inaccessibility of the dorsal surfaces of the cervical spine and their minimal bone stock.^26^ Previous biomechanical studies showed that ventral constructs show lower stiffness in flexion, compared to extension.^6^, ^18^, ^19^, ^20^ The higher peak moment of failure observed in APJ stabilization could be due to implants placed on the dorsal tension surface. The VCS group had a stiffness twice as high in extension than flexion, whereas this is inversed for the APJ stabilization. In this APJ stabilization technique, the trajectory of APJ screw placement is lateral on the neck, circumventing the deep dissection that would be necessary with a direct dorsal approach. Moreover, soft tissue dilators for a minimally invasive lateral approach using computer tomography (CT)‐guidance or computer‐assisted surgery could be considered in clinical cases, reducing the invasiveness of the ventral approach and thus complications seen with the conventional techniques.^25^, ^27^

Direct comparison with previously performed biomechanical studies is challenging due to the multiple variables (different protocols, standardizations, specimens, biomechanical testing techniques, operators, load transducers, etc). However, this study mirrored comparably designed biomechanical studies.^18^, ^19^ Biomechanical stiffness in flexion of the KCC construct (13.4–16 Nm/°), LCP (12.7–20 Nm/°) and 3D printed titanium plate and spacer (3DM) (11.9 Nm/°),^18^, ^19^, ^21^ are comparable to the PSR group in this study (11.3 Nm/°). Additionally, stiffness in extension using the KCC (18 Nm/°), LCP (19–26 Nm/°), 3DM (17 Nm/°), and PSR (22.3 Nm/°) are similar.^18^, ^19^, ^20^ Though destructive stiffness was not performed in extension in this study, the non‐destructive testing parameters showed that the bilateral APJ group had the highest stiffness in flexion and extension. In flexion, the peak moments to failure of the KCC (211 Nm), LCP (220–338 Nm), and 3DM (227 Nm) groups are comparable to the VCS group (233.03 Nm) as well as the bilateral APJ stabilization group (259 Nm). This demonstrates that bilateral APJ stabilization would likely withstand the forces seen with other implants currently used in clinical cases. The unilateral APJ stabilization group had a lower peak moment to failure than other implants,^18^, ^19^, ^20^ and was significantly lower than the VCS (i.e., PSR construct) group in this study. While exact comparisons cannot be made with historical data, in flexion and extension, the bilateral APJ group appears >2x stiffer than implants currently in use.

This study was designed principally as a proof‐of‐concept and further adjustments to these methods will be necessary before implementing this technique in horses with clinical disease. Access to the surgical site was easier in these dissected specimens than it would be expected in clinical cases where neck musculature would increase the difficulty of placement. The screw placement, straight through the APJ, might be complicated in clinical cases. Additionally, the same ventral to dorsal trajectory in this study would likely need to be adjusted for C6–C7 to avoid the shoulder to be able to accommodate placing the implants perpendicular to the APJ. We have performed bilateral APJ stabilization with a horse in lateral recumbency, alternating to achieve bilateral stabilization and we hypothesize that dorsal recumbency could permit screw insertion trajectories. Ciulu‐Angelescu et al. reports a standing, sedated approach.^17^

While cortical screws alone achieved stability in the APJs, cartilage removal and the use of spacers, bone grafting and other implant configurations require further investigation to determine ideal situations that optimize fusion prior to implementation of this technique in clinical cases.

In human medicine, clinical research of the use of facet wedges in lumbar degenerative instability has yielded successful outcomes.^28^ A modified cage or spacer placed within the APJ in conjunction with stabilization could foster bony ingrowth providing bone bridging and conferring long term stability in clinical cases. Intervertebral cages have been described in horses,^18^, ^20^ though require further investigation and adaptation to achieve APJ fusion.

Increased stiffness in non‐destructive testing of the bilateral APJ stabilization group could be due to anchoring the cervical spinal unit in two dorsal locations as opposed to a single ventral location used by other VCS implants. If the biomechanical increase in stiffness and ultimate strength of APJ stabilization can be replicated in vivo, it could improve postoperative comfort and reduce implant cycling, thereby expediting fusion and improving long‐term stability. Moreover, a combination of ventral and APJ stabilization may afford even additional stability, which could improve postoperative comfort and fusion rates, though requires further investigation.

A theoretical complication after single‐level vertebral stabilization is adjacent cervical segment deterioration through altered biomechanics and kinematics (i.e., Domino effect), though evidence of this effect occurring in horses is currently lacking. In an equine ex vivo biomechanical study, there was no increase or decrease in ROM in segments immediately cranial and caudal to the stabilized level.^29^ Further, in horses 4.4 ± 2.2 years after cervical interbody fusion with a KCC showed that there was no change in radiographic size or opacity in adjacent APJ segments after stabilization; the intervertebral disc was not evaluated.^30^

Separate biomechanical studies of the PSR (including this study), LCP and 3DM have shown APJ disarticulation after VCS.^18^, ^20^ APJ disarticulation has not been reported in vivo using any implant system, which might be explained by intact dorsal musculature removed in cadaveric biomechanical studies. Ventrally placed implants may act as a fulcrum and with tension at the intervertebral disc causes APJ distraction without catastrophic fracture. Unilateral and bilateral APJ stabilization constructs primarily failed by non‐catastrophic fracture of the cranial APJ of C5 with no cases of APJ disarticulation. If implant failure of APJ stabilization (by pull out or APJ fracture) were to occur in a clinical case, instability, discomfort, or delayed fusion could result as has been shown with VCS.^20^ However, potential complications in failure of APJ stabilization are hypothesized to be less disastrous than implant failure of the ventral stabilization and could still permit VCS techniques to be performed afterwards.^31^

The limitations of this study include the nature of an ex vivo study, sample size and unidirectional continual force to failure directly predicting in vivo conditions. Additionally, direct comparison to other VCS techniques other than PSR (KCC, LCP and 3DM) were not performed in this study. Fusion rates and implantation in living tissue require further investigation. Although not detected, there may be breed variability in anatomy and bone/soft tissue characteristics. Specimens required freezing to conduct similar testing conditions as a large number of cadavers were not available at once. For the same reason, two different cervical spinal units from the same cervical spine were used in the preliminary study. Minimal effect of freeze–thaw cycles exits on bone and intervertebral disc.^32^ Since the same number of freeze–thaw cycles were used, consistency among tested specimens would be expected.

Cyclic testing methods were not performed and thus failure modes may not represent cyclical loading that occurs in vivo. While avoiding complications directly associated with the ventral approach, a case report describing surgical repair of an articular process fracture reported transient ataxia and absence of ventilatory drive in recovery, triceps muscle swelling and suprascapular muscle atrophy and seemed to be principally caused by patient positioning.^31^ Future work will be necessary to assess the feasibility of APJ stabilization with intact external musculature, whether APJ cartilage removal is necessary to promote fusion and if periarticular callus formation occurs during fusion that could cause spinal cord compression in horses with clinical disease.

## 5 CONCLUSION

Bilateral APJ stabilization had higher biomechanical stiffness with comparable modes of failure when compared to VCS with PSR. These findings support further investigation of APJ stabilization in vivo, which may provide an alternative surgical approach to promote spinal fusion in horses.

## AUTHOR CONTRIBUTIONS

Ysebaert MP, BVetSci, MVetMed, MS: Provided substantial contributions of the study design, collected the cadaveric specimens, prepared the cervical spinal units, performed the surgical procedures, performed data acquisition and analysis, drafted the manuscript and revised it critically and approved the final version. Foslien R, BS: Provided substantial contributions to biomechanical study design, collected and analyzed biomechanical data, revised the draft and approved the final version. David DM, DVM: Aided in collection and preparation of cervical spine units. Bonilla AF, MVZ, MSc, PhD: Provided contributions to the study design and acquisition of data, critically revised the draft and approved the final version. Gadomski BC, PhD: Aided in design of the study, provided scientific, in‐line editing of the manuscript. Easley JT, DVM, DACVS (Large Animal): Aided in interpretation of the results, provided scientific, in‐line editing of the manuscript. Nelson BB, DVM, PhD, DACVS (Large Animal): Contributed to design of the study, aided in surgical procedures, analyzed the data for statistical significance, and provided scientific, in‐line editing of the manuscript as the senior author. All authors provided a critical review of the manuscript and endorse the final version. All authors are aware of their respective contributions and have confidence in the integrity of all contributions.

## CONFLICT OF INTEREST STATEMENT

The authors declare no conflict of interest related to this report.

## Data Availability

Study data is available upon request by contacting the corresponding author.
